# New Onset Multiple Sclerosis Post-COVID-19 Vaccination and Correlation With Possible Predictors in a Case-Control Study

**DOI:** 10.7759/cureus.36323

**Published:** 2023-03-18

**Authors:** Majed Alluqmani

**Affiliations:** 1 Neurology, Taibah University, Medina, SAU

**Keywords:** covid-19 vaccines, case control study, neuromyelitis, nervous system, multiple sclerosis

## Abstract

Introduction: Various inflammatory diseases have been associated with the administration of various vaccines. Several reports have associated vaccine administration with the demyelinating diseases of the central nervous system (CNS). However, no clear or strong scientific evidence exists to support the association of vaccine administration with the onset of demyelinating diseases. Some CNS demyelination diseases such as acute disseminated encephalomyelitis (ADEM) and neuromyelitis optica spectrum disorders (NMOSD) were reported following the administration of COVID-19 vaccines. In this study, new onset multiple sclerosis (MS) following COVID-19 vaccine administration was reported.

Methods: In this longitudinal observational case-control study, a total of 65 participants were studied, who were divided into two groups. Group A included 32 MS patients who were diagnosed post-COVID-19 vaccine administration and group B included 33 participants who received COVID-19 vaccines and did not develop MS. Group B was used as a control. The Chi-square test and logistic regression analysis were carried out using Statistical Product and Service Solutions (SPSS) (IBM SPSS Statistics for Windows, Armonk, NY) software.

Results: Univariate and multivariate logistic regression analysis was performed and a significant correlation between the risk factors and the development of MS post-COVID-19 vaccination was identified.

Conclusion: The risk factors, identified in this study, can be used as significant independent predictors for developing MS post-COVID-19 vaccinations.

## Introduction

A wide variety of inflammatory diseases has been associated with the administration of various vaccines. Concerns that demyelinating diseases of the central nervous system (CNS) might trigger after vaccine administration have existed for a long time [1]. However, most of these concerns were case reports [2]. There has been no clear or strong scientific evidence to support the association of vaccine administration with demyelinating disease onset specifically CNS demyelinating diseases [3]. Vaccines against the influenza viruses have been widely reported as vaccinations associated with CNS demyelinating diseases. Moreover, other vaccines such as human papillomavirus (HPV), hepatitis A or B, rabies, measles, rubella, yellow fever, anthrax, meningococcus, and tetanus have also been reported as a trigger for post-vaccination CNS demyelinating diseases [4]. Odds ratios (OR) of associations between ever having vaccination and risk of demyelinating diseases (MS) were 0.9 (0.6-1.5) for HBV, 0.6 (0.4-0.8) for tetanus vaccination, 0.8 (0.5-1.5) for influenza vaccine, 0.8 (0.5-1.5) for measles, mumps, and rubella (MMR), 0.9 (0.5-1.4) for measles vaccine, 0.7 (0.4-1.0) for rubella [2].

Vitamin D is a well-known risk factor as well as a prognostic factor for the development of MS [5]. Moreover, recently an association between Epstein-Barr virus (EBV) and the development of multiple sclerosis (MS) has also been reported [6].

In 2019, the coronavirus disease (COVID-19) pandemic, caused by severe acute respiratory syndrome coronavirus 2 (SARS-CoV-2), caused great damage to the global economy, and social life worldwide. At the end of 2020, emergency approval for the use of COVID-19 vaccines had been granted by the World Health Organization (WHO) to different research laboratories around the world. Currently, four main types of vaccines against COVID-19 are approved. These types are whole virus (live attenuated, inactivated), nucleic acid (mRNA, DNA), viral vector (non-replicating, replicating), and protein-based (subunit, virus-like particle) vaccines [7].

CNS demyelination diseases such as acute disseminated encephalomyelitis (ADEM) and neuromyelitis optica spectrum disorders (NMOSD) were reported by different health organizations following the administration of all types of approved COVID-19 vaccines. However, only a few patients were newly diagnosed with MS following vaccine administration [8].

This study aimed to illustrate the prevalence of new-onset MS post-COVID-19 vaccination and correlate it with its possible predictors in a multi-center case-control study.

## Materials and methods

Patient population

This longitudinal observational case-control study has included a total of 65 participants. Participants were divided into two groups. Group A included 32 MS patients who were diagnosed post-COVID-19 vaccination and group B included 33 participants who received COVID-19 vaccinations and did not develop MS. Group B was used as a control group. The required demographic and clinical data have been collected from neurology clinic databases in King Fahd Hospital (KFH) and Saudi German Hospital (SGH) at Al Madinah Al Munawwarah in the Kingdom of Saudi Arabia. All studied patients are Arabs. The patients were thoroughly examined and were followed up for one year in outpatient clinics by the author. The research ethics committee of the college of medicine, Taibah University Medina provided ethical approval for the study. The IRB approval number is CM-REC 036-1441.

Collection of clinical data

All participants were subjected to detailed clinical examination. Complete medical history, general and local neurological examination, and radiological work including magnetic resonance imaging (MRI) with contrast on the brain and spinal cord were carried out. Clinical examination was performed at the time of symptom presentation as well as on follow-up after one year of onset. Also, they were sampled for routine blood laboratory tests including serum 25-hydroxy vitamin D. Vitamin D concentration of 30 ng/ml 25(OH)D is considered sufficient, with values between 29 and 20 ng/ml as insufficiency, and patients with levels less than 20 ng/ml were considered deficient [9]. Moreover, participants were also sampled for Epstein-Barr nuclear antigen 1 (EBNA-1) IgG, while all participants who developed symptoms suggestive of demyelinating CNS disease were sampled for cerebrospinal fluid (CSF) oligoclonal bands (OCB) and other laboratory investigations for MS mimics. The patients who developed MS post-COVID-19 vaccinations were followed for functional disability after one year using an expanded disability status scale (EDSS) and imaging. All data were collected and subjected to statistical analysis using univariate and multivariate logistic regression analysis.

## Results

Demographics

Among 65 study participants, there were 45 females and 20 males. Ages ranged between 21 and 45 years with a median age of 30 years. The case-control group did not experience any neurological manifestations post-COVID-19 vaccinations, while the neurological presentation symptoms among the MS group showed that vertigo and imbalance symptoms were the most commonest (21 patients), then visual symptoms (seven patients), and the least common were motor symptoms (three patients).

Onset of MS and vaccine type

The time of onset of these symptoms post-COVID-19 vaccinations ranged between one and two months with a median time of two months. Among case-control groups, 12 have received the Pfizer vaccine, six have received the Moderna vaccine, and 15 have received the AstraZeneca vaccine. Among MS, 20 have received the Pfizer vaccine, three have received the Moderna vaccine, and nine have received the AstraZeneca vaccine. The presentation of symptoms among the MS group appeared after the first dose of vaccine in 25 patients, while it appeared after the second dose of vaccine in seven patients (Table 1).

**Table 1:** Comparison between the two studied groups according to different parameters SD: standard deviation; PFIZ: Pfizer; MOD: Moderna; ASZ: AstraZeneca p: p value for comparing between Non-MS and MS *: Statistically significant at p ≤ 0.05

	Total (n=65)	Non-MS (n=33)	MS (n=32)	Test of sig.	P
	No. (%)	No. (%)	No. (%)		
Gender					
Male	20 (30.8%)	11 (33.3%)	9 (28.1%)		0.649
Female	45 (69.2%)	22 (66.7%)	23 (71.9%)		
Age (years)					
Median (Min.-Max.)	30 (21-45)	29 (21-45)	30.5 (21-45)	0.199	0.843
Mean ± SD	31.3 ± 7	31.2 ± 7	31.5 ± 7.2		
Presentation symptoms					
No	33 (50.8%)	33 (100%)	0 (0%)		<0.001^*^
Vertigo	11 (16.9%)	0 (0%)	11 (34.4%)		
Imbalance	1 (1.5%)	0 (0%)	1 (3.1%)		
Sensory	10 (15.4%)	0 (0%)	10 (31.3%)		
Visual	7 (10.8%)	0 (0%)	7 (21.9%)		
Motor	3 (4.6%)	0 (0%)	3 (9.4%)		
Time onset post-vaccination (months)					
Median (Min.-Max.)	2 (1-5)	–	2 (1-5)		–
Mean ± SD	2.4 ± 1.2	–	2.4 ± 1.2		
Vaccine type					
PFIZ	32 (49.2%)	12 (36.4%)	20 (62.5%)	4.440^*^	0.035^*^
MOD	9 (13.8%)	6 (18.2%)	3 (9.4%)	1.056	0.475
ASZ	24 (36.9%)	15 (45.5%)	9 (28.1%)		0.148
Presentation of symptoms after vaccine doses					
No	33 (50.8%)	33 (100.0%)	0 (0.0%)	80.071^*^	<0.001^*^
First	25 (38.5%)	0 (0.0%)	25 (78.1%)		
Second	7 (10.8%)	0 (0.0%)	7 (21.9%)		

MRI outcomes after vaccine administration

Among the case-control group, there were neither MRI brain nor spinal cord lesions at any time during our study, while in the MS group, there were brainstem demyelinating lesions in 14 patients, cerebral demyelinating lesions in 12 patients, cervical spinal cord lesions in six patients, and dorsal spinal cord lesions in two patients. MRI of the brain and spinal cord with contrast were also carried out after one year during follow-up and showed no new demyelinating lesions among the control group (Group B), while there were new demyelinating lesions in 18 patients among the MS group (Group A). Serum level of vitamin D was low in 12 participants of the control group, while it was low in 22 patients of the MS group. EBNA1-IgG was positive in 12 participants of the control group, while it was positive in 22 patients in the MS group. In the control group, there were 10 smoker participants, four participants with a family history of MS, and four participants with a history of the previous immune-mediated disease, while in the MS group, there were 13 smoker patients, 11 patients with a family history of MS, and 10 patients with a history of the previous immune-mediated disease (Table 2).

**Table 2:** Comparison between the two studied groups according to different parameters SD: standard deviation; MRI: magnetic resonance imaging; EDSS: expanded disability status scale; CSF: cerebrospinal fluid p: p value for comparing between Non-MS and MS *: Statistically significant at p ≤ 0.05

	Total (n=65)	Non-MS (n=33)	MS (n=32)	Test of sig.	P
	No. (%)	No. (%)	No. (%)		
MRI brain lesions					
No	39 (60.0%)	33 (100.0%)	6 (18.8%)	44.688^*^	<0.001^*^
Brainstem	14 (21.5%)	0 (0.0%)	14 (43.8%)		
Cerebral	12 (18.5%)	0 (0.0%)	12 (37.5%)		
MRI spinal cord lesions					
No	57 (87.7%)	33 (100%)	24 (75%)	9.091^*^	0.002^*^
Cervical	6 (9.2%)	0 (0%)	6 (18.8%)		
Dorsal	2 (3.1%)	0 (0%)	2 (6.3%)		
MRI brain and spinal cord follow-up after one year					
No new lesions	47 (72.3%)	33 (100.0%)	14 (43.8%)	25.672^*^	<0.001^*^
New lesions	18 (27.7%)	0 (0.0%)	18 (56.3%)		
EDSS score after one year					
Median (Min.-Max.)	0 (0-2)	0 (0-0)	1 (0-2)	231.0^*^	<0.001^*^
Mean ± SD	0.37 ± 0.64	0 ± 0	0.75 ± 0.74		
CSF oligoclonal bands					
Absent	47 (72.3%)	33 (100.0%)	14 (43.8%)	25.672^*^	<0.001^*^
Present	18 (27.7%)	0 (0.0%)	18 (56.3%)		
Investigations for multiple sclerosis mimics					
Negative	65 (100.0%)	33 (100.0%)	32 (100.0%)		–
Serum level of vitamin D ng/ml					
Low	34 (52.3%)	12 (36.4%)	22 (68.8%)	6.831^*^	0.009^*^
Average	31 (47.7%)	21 (63.6%)	10 (31.3%)		
EBNA1-IgG					
Negative	31 (47.7%)	21 (63.6%)	10 (31.3%)	6.831^*^	0.009^*^
Positive	34 (52.3%)	12 (36.4%)	22 (68.8%)		
Smoking					
No	42 (64.6%)	23 (69.7%)	19 (59.4%)	0.757	0.384
Yes	23 (35.4%)	10 (30.3%)	13 (40.6%)		
Family history of multiple sclerosis					
No	50 (76.9%)	29 (87.9%)	21 (65.6%)	4.532^*^	0.033^*^
Yes	15 (23.1%)	4 (12.1%)	11 (34.4%)		
History of previous immune-mediated disease					
No	51 (78.5%)	29 (87.9%)	22 (68.8%)	3.518	0.061
Yes	14 (21.5%)	4 (12.1%)	10 (31.3%)		

**Figure 1:**
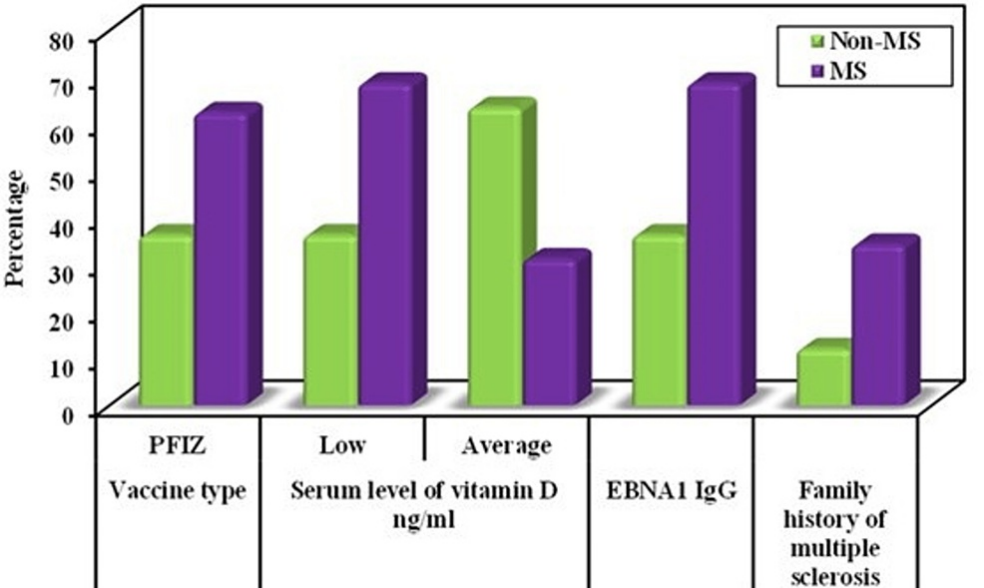
Comparison between the two studied groups according to different parameters PFIZ: Pfizer; MS: multiple sclerosis

This study showed that there were significant correlations between the following risk factors and developing MS post-COVID-19 vaccination using univariate and multivariate logistic regression analysis: Pfizer vaccine (P value 0.040), low serum level of vitamin D (P value 0.015), Positive EBNA1-IgG (P value 0.027), and family history of MS (P value 0.043). These risk factors can be used as significant independent predictors for developing MS post-COVID-19 vaccinations (Table 3 and Figure 1).

**Table 3:** Univariate and multivariate logistic regression analysis for the different risk factors of MS (n =32) OR: odd`s ratio; C.I: confidence interval; LL: lower limit; UL: upper limit; PFIZ: Pfizer; MOD: Moderna; ASZ: AstraZeneca #: All variables with p<0.05 were included in the multivariate *: Statistically significant at p ≤ 0.05

	Univariate		^#^Multivariate	
	P	OR (LL-UL 95% C.I)	p	OR (LL-UL 95% C.I)
Female	0.650	1.278 (0.444-3.678)		
Age (years)	0.840	1.007 (0.939-1.080)		
Vaccine type				
PFIZ	0.037^*^	2.917 (1.065-7.989)	0.040^*^	3.542 (1.060-11.838)
MOD	0.312	0.466 (0.106-2.048)		
ASZ	0.151	0.470 (0.167-1.317)		
Serum level of vitamin D ng/ml	0.010^*^	0.260 (0.093-0.728)	0.015^*^	0.223 (0.066-0.748)
EBNA1-IgG	0.010^*^	3.850 (1.374-10.789)	0.027^*^	3.824 (1.169-12.515)
Smoking	0.386	1.574 (0.565-4.382)		
Family history of multiple sclerosis	0.040^*^	3.798 (1.061-13.587)	0.043^*^	4.511 (1.050-19.380)
History of previous immune-mediated disease	0.069	3.295 (0.912-11.914)		

## Discussion

WHO had suggested certain criteria to define vaccination-induced entities [10]. These criteria include (A) temporal relationship (vaccination must precede the occurrence of the event, (B) consistency of evidence (equivalent results reported by studies using different methods in different settings), (C) strength of association (statistical significance), (D) specificity (vaccine is the only cause of the event), (E) biological plausibility and coherence. It is hard to meet these criteria which have made it a challenge to define the majority of cases in the literature including our case as a vaccination-induced entity. In this longitudinal observational case-control study, the above criteria have been applied, and I was able to give a diagnosis of new-onset relapsing-remitting MS post-COVID vaccination for 32 patients. These patients have developed their first MS attack after two to five weeks of COVID-19 vaccinations (all four types of approved vaccines). Over the next year, clinical and radiological follow-ups have been done for these patients and a diagnosis of MS has been confirmed based on MacDonald criteria 2017.

Furthermore, in this study, patients were grouped according to their clinical presentation and similarity among MS known risk factors. Based on that, females gender in their 30s were more susceptible. Central vertigo, cerebral sensory symptoms (tingling and numbness), and visual symptoms (double vision and optic neuritis) were the most common presenting symptoms among these patients. In terms of the second attack (either clinical or active radiological or both), it mostly occurred within two months post-first dose of mRNA COVID vaccine. Also, a one-year follow-up MRI showed that the brain stem lesion was the most common location involvement among our patients during the first presentation, and new lesions were detected either in the brain or cervical spine.

MS is a chronic demyelinating disease of the CNS. Genetic and environmental factors are believed to play a role as risk factors for MS. The ability of vaccines to either cause or exacerbate MS has been evaluated in several studies such as hepatitis B vaccine, tetanus, or influenzas vaccines. However, pooled analysis from multiple studies found no clear evidence to support a causal relationship between the onset of MS and vaccinations. Moreover, recent systematic reviews found no clear evidence of an increased risk of developing MS and in-relapses after vaccination [11,12].

Cases of post-COVID-19 demyelinating disease have been reported, however, few cases with new MS onset post-COVID vaccine have been reported to date. In terms of COVID-19, Achiron and colleagues report no increased risk of relapse activity in MS patients who received the BNT162b2 vaccine, and the relapse rate was higher following the first dose than the second dose in younger patients [13,14]. To the best of our knowledge, few patients (six patients) with de novo MS diagnosis following recent COVID-19 vaccination has been published so far [15,16]. These cases are closely similar to our patients with regards to the time of onset post-vaccine, more with mRNA type vaccine and the clinical course as well.

Limitations

This study has some limitations within which the findings need to be interpreted. An insufficient sample size might have effects on the statistical measurements. Moreover, this study has been performed in Arab populations and the results cannot be generalized.

## Conclusions

In this study, it is proposed that the COVID vaccine can trigger the immune system which helps with self-antigens production of CNS in susceptible people. Also, this study showed that there were significant correlations between the following risk factors and developing MS post-COVID-19 vaccinations: Pfizer vaccine, low serum level of vitamin D, positive EBNA1-IgG, and family history of MS. These risk factors can be used as significant independent predictors for developing MS post-COVID-19 vaccinations. However, genetic and environmental factors cannot be ruled out. Furthermore, in this study, a definite association between COVID-19 vaccine with MS is not described, rather it is aimed to highlight the possibility of this association as an independent risk factor that can trigger new onset MS in the susceptible group. Though, more data is needed for further correlation.
